# First record of two Copepoda species parasitizing *Colomesus tocantinensis* (Tetraodontiformes: Tetraodontidae) in the Tocantins-Araguaia basin, Brazil

**DOI:** 10.1590/S1984-29612023030

**Published:** 2023-05-29

**Authors:** Gabriela Michelan, Wagner Toshio Hasuike, Lidiany Doreto Cavalcanti, Atsler Luana Lehun, João Otávio Santos Silva, Ricardo Massato Takemoto

**Affiliations:** 1 Programa de Pós-graduação em Biologia Comparada – PGB, Universidade Estadual de Maringá – UEM, Maringá, PR, Brasil; 2 Núcleo de Pesquisa em Limnologia, Ictiologia e Aquicultura – NUPELIA, Universidade Estadual de Maringá – UEM, Maringá, PR, Brasil; 3 Programa de Pós-graduação em Ecologia de Ambientes Aquáticos Continentais – PEA, Universidade Estadual de Maringá – UEM, Maringá, PR, Brasil

**Keywords:** Pufferfish, Lernaeidae, Ergasilidae, Legal Amazon, fish parasites, Baiacu, Lernaeidae, Ergasilidae, Amazônia Legal, parasitas de peixe

## Abstract

Considering the lack of studies on freshwater fishes of the genus *Colomesus*, we conducted a survey the parasite fauna of *Colomesus tocantinensis* collected from the Tocantins River, Brazil. We first recorded the presence of the ectoparasites *Ergasilus colomesus* and *Lernaea* sp. where 96.77% of the fish were parasitized.

## Resumo

Considerando a ausência de estudos com os peixes de água doce do gênero *Colomesus,* foi realizado um levantamento da parasitofauna de *Colomesus tocantinensis* coletados no rio Tocantins. Foi registrada pela primeira vez a presença dos ectoparasitas *Ergasilus colomesus* e *Lernaea* sp. onde 96,77% dos peixes estavam parasitados.

The Tocantins River is composed of the Tocantins-Araguaia basin and is located in the Legal Amazon. It originates in the state of Goiás and runs northward for 2500 km, passing through the states of Tocantins, Maranhão, and Pará (Santos et al., 2004). The Tocantins-Araguaia basin is known to drain part of the extreme south of the Brazilian Shield to the extreme north of the Amazon basin, making the ichthyofauna of both basins closely related and presenting a high degree of endemism and diversity (Goulding et al., 2003; Lucinda et al., 2007; Bertaco & Carvalho, 2010; Amaral et al., 2013).

The family Tetraodontidae is composed of 192 fish species, distributed in 28 genus that occur in the seas, estuaries, and rivers of tropical and temperate regions (Fricke et al., 2023). The genus *Colomesus* comprises only three fish species, two of which are freshwater: *Colomesus asellus* (Müller & Troschel, 1849) and *C. tocantinensis* Amaral et al., 2013, both endemic to South America and distributed throughout the Amazon and Tocantins-Araguaia basins, respectively (Amaral et al., 2013).

These animals, known as “pufferfish,” can inflate their bodies in stressful situations and are coveted by the ornamental fish industry. The two species have similar morphological characteristics; however, their feeding habits have only been recorded in studies conducted with *C. asellus* (Bartolette et al., 2018). The same is observed for the parasitic fauna of these hosts; thus far, only the ectoparasite *E. colomesus* Thatcher and Boeger, 1983 has been found in the gills of *C. asellus* (Thatcher & Boeger, 1983; Virgilio et al., 2021), whereas for *C. tocantinensis* there are no parasitological records. Considering the absence of biological and ecological studies of *C. tocantinensis*, we present the first parasitological survey of this species.

Thirty-one individuals of *C. tocantinensis* were collected with nets of different mesh sizes from the Tocantins River, city of Porto Nacional (10°42'25.9 “S, 48°25'14.0 “W) in 2018. The procedures for necropsy of the hosts and collection, preservation, and preparation of parasites were performed according to the methods reported by Eiras et al. (2006). Parasitological indices were calculated according to Bush et al. (1997). Representative host specimens were deposited in the Ichthyological Collection of Universidade Federal de Tocantins (UNT 20349).

**Figure 1:**
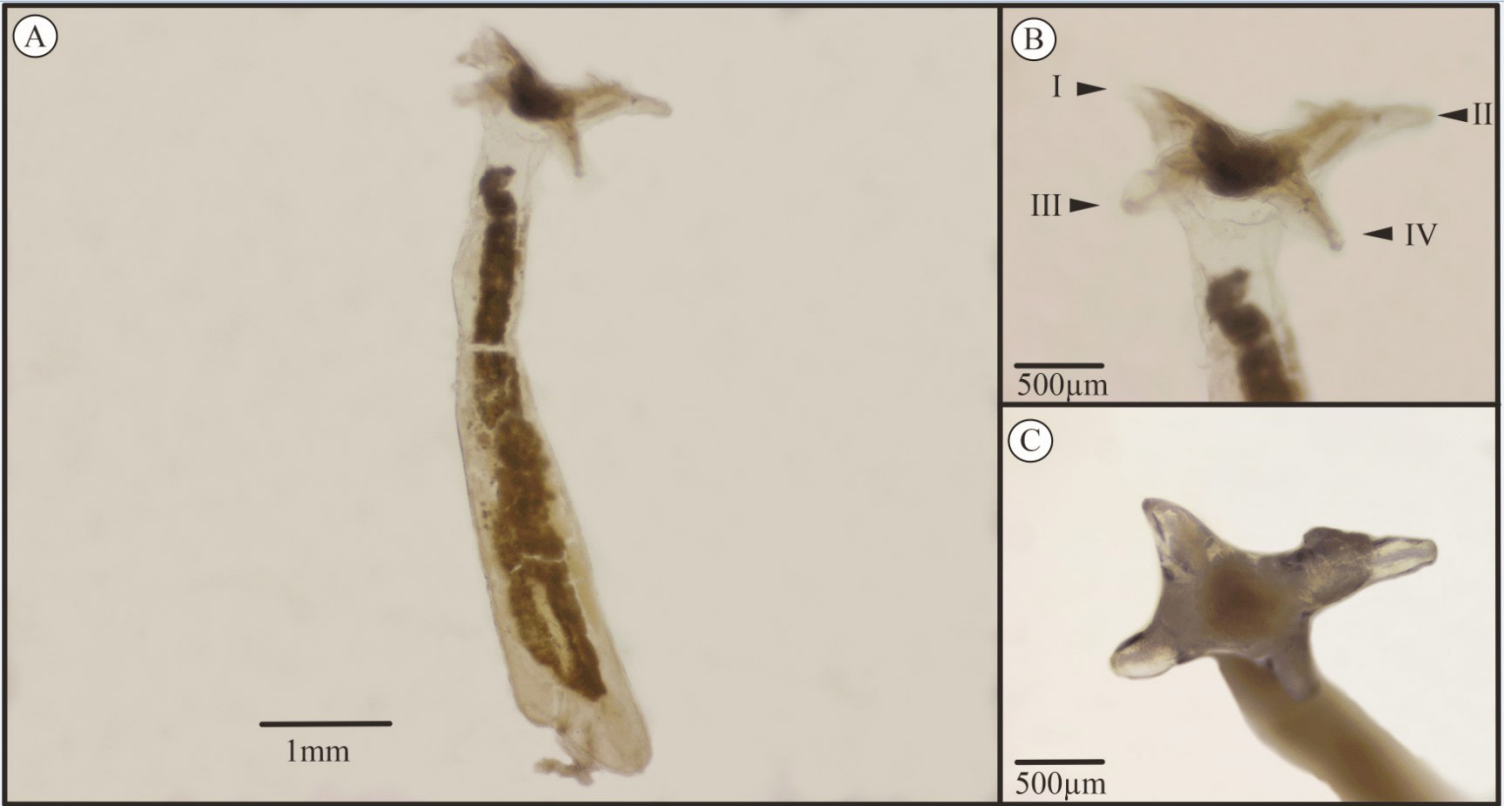
(A) *Lernaea* sp. parasite of *Colomesus tocantinensis*; (B) Anchor (arrows I, II, III and IV: holdfast); (C) Apical view of anchor attachment.

**Figure 2:**
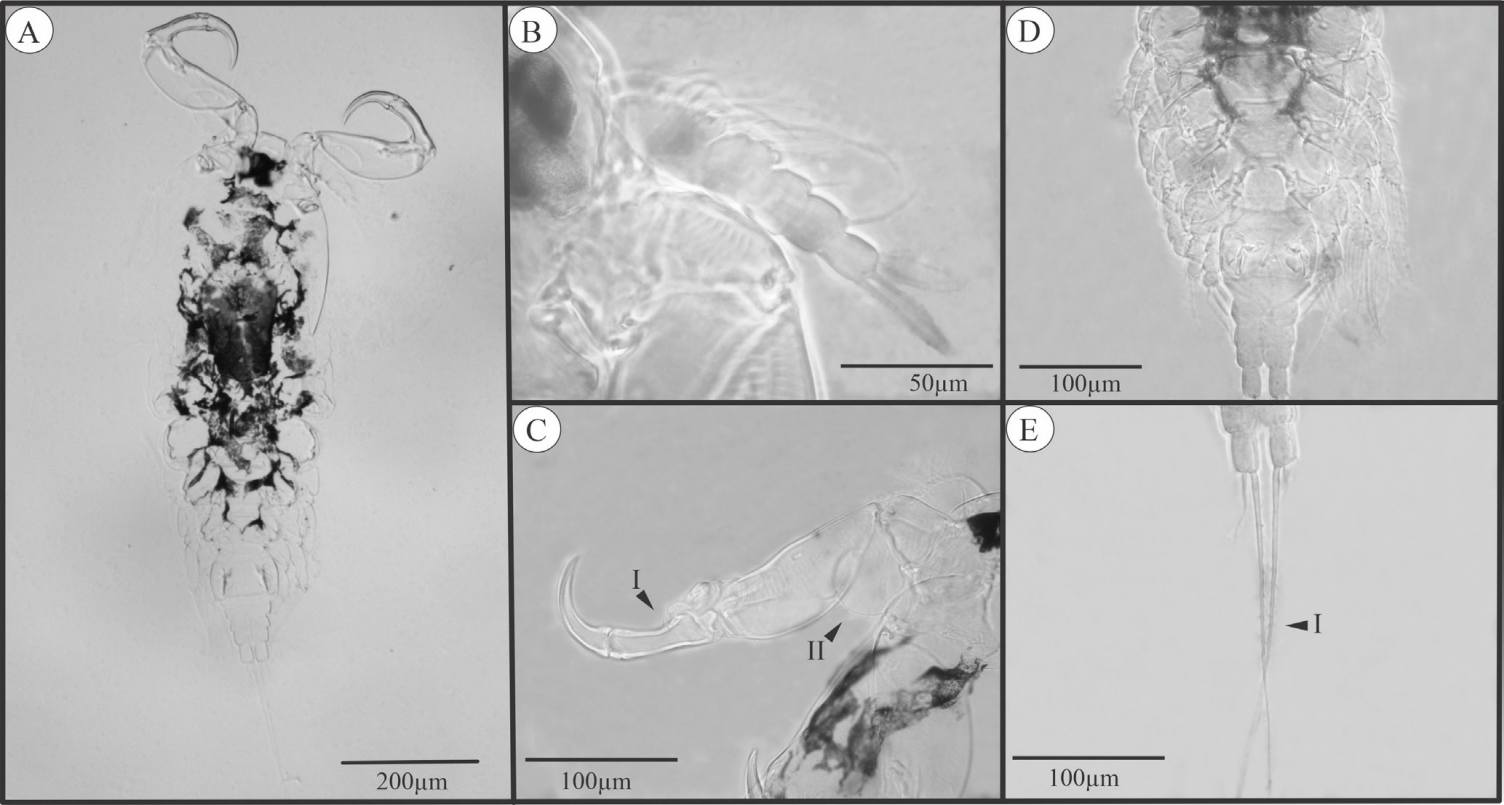
(A) *Ergasilus colomesus* parasite of *Colomesus tocantinenses* (dorsal view); (B) First antenna; (C) Modified second antenna (Arrow I: Spine; Arrow II: Laterally inflated anther basal segment; (D) Legs; (E) Uropod (Arrow I: Long, sparsely hairy bristle).

Thirty of the analyzed hosts were parasitized by at least one parasite. We recorded, for the first time, the interaction of *Lernaea* sp. (Figure 1) and *E. colomesus* (Figure 2) with *C. tocantinensis*. One *Lernaea* sp. specimen was found in only one individual, and the parasite with the highest frequency of occurrence, abundance, and mean intensity was *E. colomesus*, which was the central parasite for the host and had a high prevalence (Table 1). In a study describing the interaction of this parasite with *C. asellus*, prevalence was also high as the parasite was present in 76% of the hosts collected (Thatcher & Boeger, 1983). However, in a study conducted by Virgilio et al. (2021), the prevalence of *E. colomesus* in individuals of *C. asellus* was low and varied between the dry and flood periods, with values of 37.5% and 23.8%, respectively.

**Table 1:** Prevalence (%), Mean Intensity and Mean Abundance of ectoparasites of *Colomesus tocantinensis* from the Tocantins-Araguaia basin collected in the Tocantins River.

Parasites	Prevalence (%)	Mean Intensity (±SE)	Mean Abundance (±SE)
Copepoda			
*Ergasilus colomesus*	96.77	8.56±1.90	8.29±1.86
*Lernaea* sp.	3.22	1±1	0.03±1

SE: Standard error.

The family Ergasilidae, in general, does not exhibit high specificity; however, through evaluation of the species of the genus *Ergasilus* in isolation, several records of parasites restricted to a single host can be varified (Luque et al., 2013). We found that although the two *Colomesus* species inhabit different basins, the *E. colomesus* parasite maintained specificity at the host genus level. In general, ectoparasites do not require intermediate hosts to complete their life cycle. In the case of copepods, the initial stages (nauplii and copepodites) are freeborn, and after actively infecting the host, they undergo metamorphosis until they reach the adult stage and reproduce (Ohtsuka et al., 2018). These characteristics (specificity and reproduction) may explain the presence of copepods in *C. tocantinensis*.

The absence of endoparasites recorded in this study can be explained by the biology of the “pufferfish,” as these fish have three defense mechanisms: being venomous, inflation (with water or air), and aposematism (dorsal transverse black bars as warning coloration) (Krebs & Davis, 1993), which can prevent predation. Thus, from an endoparasitic perspective, non-host species that consume infected intermediate hosts are a ‘dead end’ and cannot act as competent final hosts to complete their life cycle (Mouritsen & Poulin, 2003; Song & Proctor, 2020). Considering that endoparasites primarily use the trophic pathway to access their hosts (Lafferty et al., 2008), and that there is no influence of ontogeny or hydrometric period on the feeding of *C. tocantinensis* individuals (Bartolette et al., 2018), it is unlikely that the absence of endoparasites is due to collection bias or sampling number, as these variables would not inhibit infection.

Our study provides new insights on the parasitic fauna of *C. tocantinensis*, analyzing for the first time the record of two species of ectoparasites (*Lernaea* sp. and *E. colomesus*) for this host. We emphasize the need for further study, as existing studies on the biology and ecology of this species are limited.
